# The conserved *ASTN2*/*BRINP1* locus at 9q33.1–33.2 is associated with major psychiatric disorders in a large pedigree from Southern Spain

**DOI:** 10.1038/s41598-021-93555-4

**Published:** 2021-07-15

**Authors:** Josep Pol-Fuster, Francesca Cañellas, Laura Ruiz-Guerra, Aina Medina-Dols, Bàrbara Bisbal-Carrió, Bernat Ortega-Vila, Jaume Llinàs, Jessica Hernandez-Rodriguez, Jerònia Lladó, Gabriel Olmos, Konstantin Strauch, Damià Heine-Suñer, Cristòfol Vives-Bauzà, Antònia Flaquer

**Affiliations:** 1 Department of Biology, University of Balearic Islands (UIB), Institut Universitari d’Investigacions en Ciències de la Salut (IUNICS), Palma, Spain; 2 Neurobiology Laboratory, Research Unit, Son Espases University Hospital (HUSE), Health Research Institute of Balearic Islands (IdISBa), Floor -1, Module F, R-805 Palma, Spain; 3 Department of Psychiatry, HUSE, IdISBa, Palma, Spain; 4 Molecular Diagnostics and Clinical Genetics Unit (UDMGC) and Genomics of Health Research Group, Hospital Universitari Son Espases (HUSE) and Institut d′Investigacions Sanitaries de Balears (IDISBA), Palma, Spain; 5 Institute of Medical Biostatistics, Epidemiology and Informatics (IMBEI), University Medical Center, Johannes Gutenberg University, Mainz, Germany; 6 Institute of Genetic Epidemiology, Helmholtz Zentrum München-German Research Center for Environmental Health, Neuherberg, Germany; 7 Institute of Medical Informatics, Biometry and Epidemiology, Chair of Genetic Epidemiology, LMU Munich, Munich, Germany

**Keywords:** Genetics, Behavioural genetics, Genetic linkage study, Genomics, Neurodevelopmental disorders

## Abstract

We investigated the genetic causes of major mental disorders (MMDs) including schizophrenia, bipolar disorder I, major depressive disorder and attention deficit hyperactive disorder, in a large family pedigree from Alpujarras, South of Spain, a region with high prevalence of psychotic disorders. We applied a systematic genomic approach based on karyotyping (n = 4), genotyping by genome-wide SNP array (n = 34) and whole-genome sequencing (n = 12). We performed genome-wide linkage analysis, family-based association analysis and polygenic risk score estimates. Significant linkage was obtained at chromosome 9 (9q33.1–33.2, LOD score = 4.11), a suggestive region that contains five candidate genes *ASTN2*, *BRINP1*, *C5*, *TLR4* and *TRIM32,* previously associated with MMDs. Comprehensive analysis associated the MMD phenotype with genes of the immune system with dual brain functions. Moreover, the psychotic phenotype was enriched for genes involved in synapsis. These results should be considered once studying the genetics of psychiatric disorders in other families, especially the ones from the same region, since founder effects may be related to the high prevalence.

## Introduction

Psychiatric disorders aggregate in families and their predisposition involve a complex, polygenic and pleiotropic genetic architecture^1–3^. Patterns of shared genetic material have shown across the five major mental disorders (MMD): autism spectrum disorder (ASD), schizophrenia (SCZ), bipolar disorder (BD), major depressive disorder (MDD) and alcoholism^1–5^. Genetic epidemiological studies have revealed that the risk of developing one of these disorders is proportional to the genomic material shared with an affected individual^6^. In fact, the heritability of MMDs has been estimated as being at least 80%^6,7^. Thanks to the application of whole-genome scan technologies, as genome-wide association studies (GWASs) and next generation sequencing, in the recent years we have observed a dramatic improvement in identifying genetic risk factors for these disorders^8–11^. Of those, common SNPs have shown to contribute to around 20% of the heritability, with individually weaker contributions (odds ratios, < 1.2)^12^. Meanwhile, copy number variants (CNVs) as well as rare de novo or recent single-nucleotide variants (SNVs) have evidenced higher impacts (odds ratios, 2–57)^13,14^. The challenge now resides in applying these technologies to establish personalized diagnoses. The Psychiatric Genomic Consortium (PGC) in his latest published agenda aims to study large pedigrees to search for genetic variants of large effect^15^. Pedigrees from genetic isolates with high degrees of consanguinity are of special interest. Several large pedigrees have been recently investigated, either looking for CNVs^16–18^, rare SNVs^19–24^ or common variant contributions^25^. But very few have followed the PGC suggestions^15^, aimed to analyze those pedigrees using comprehensive genomic assays^26,27^.

In this study we have applied a systemic genomic approach to uncover the genomic architecture of a large lineage, with 41 individuals affected of MMD in the last three generations, 27 of which have been diagnosed with psychotic disorders. This family is from a region of southern Spain, the Alpujarras, known to be a hotspot for psychiatric diseases, with a prevalence of 7.8%^28^, almost double of that from the rest of the country, suggestive of being due to founding genetic events.

## Results

### Pedigree description

**Figure 1:**
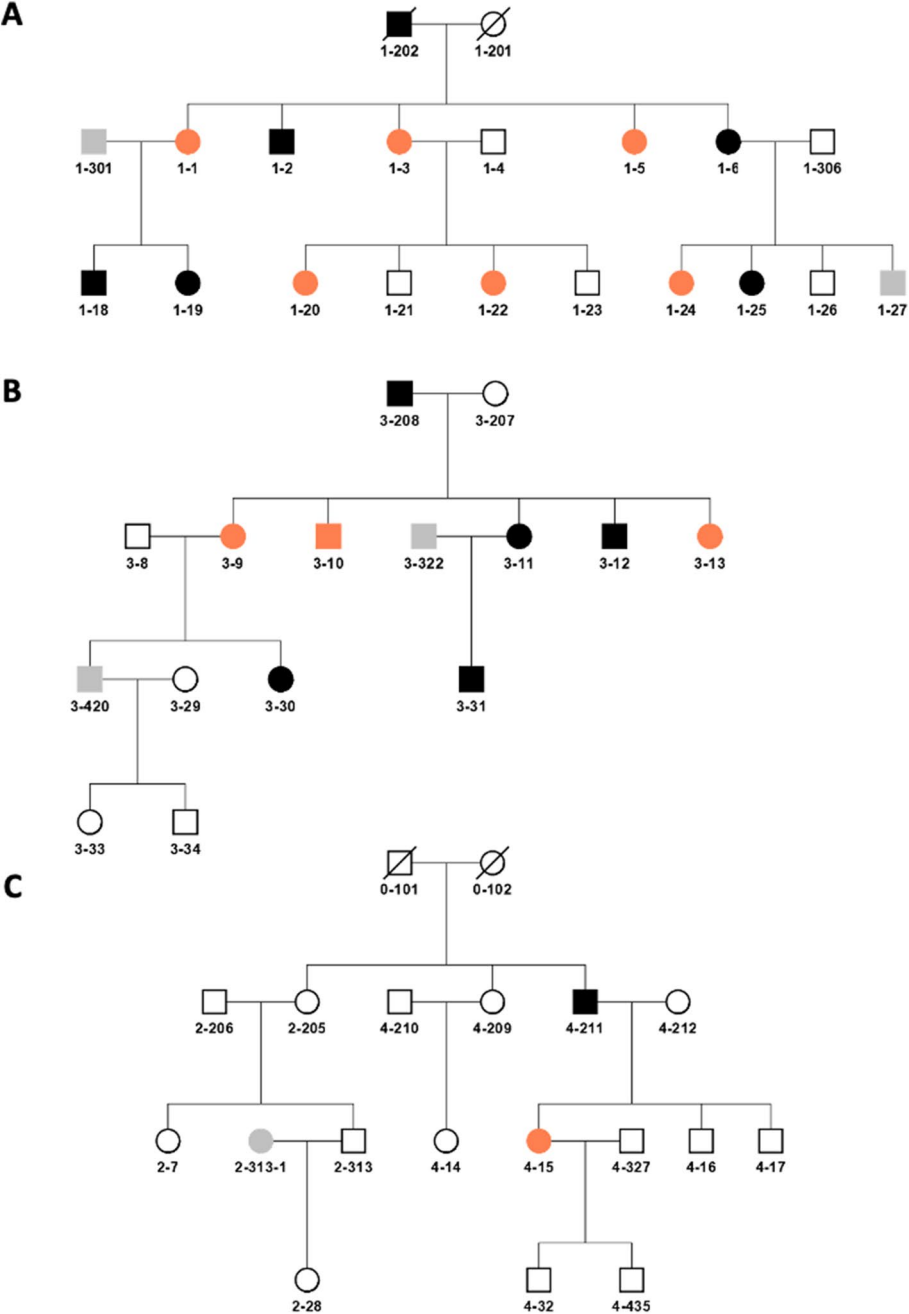
Pedigree structures of the three subfamilies analyzed. Subfamilies 1 (**A**), 3 (**B**) and 2 (**C**). DNA was available for all the subjects numbered from 1 till 35. Black indicates a diagnosis of psychosis, comprising SCZ, SCA and BD-I. Orange indicates a diagnosis of mental disorder without psychosis, comprising MDD and ADHD. Grey indicates undetermined diagnosis.

A large multigenerational family of Southern Spanish origin with high prevalence of mental disorder was recruited between the Psychiatry ward of the University Hospital Son Espases (HUSE) of the Balearic Islands and the Health Center of El Ejido. The full pedigree is shown in Fig. S1. Figure 1 shows the three subfamilies analyzed. Subjects 1–202 (subfamily 1), 4-211 (subfamily 2) and 3–208 (subfamily 3) are siblings. Out of the 41 individuals affected of MMD, 27 have been diagnosed with psychosis and 14 with a mental disease without psychosis. A clinical description of the family subjects is summarized in Table S1, showing the Global Assessment of Functioning (GAF) scale for all psychotic subjects studied and the Positive and Negative Syndrome Scale (PANSS) scores for all the schizophrenic patients analyzed (Table S1).

#### Phenotype definition

In order to perform the genomic analysis, two phenotypes were defined: the narrow phenotype was attributed only to patients with psychosis (n = 27), including: SCZ, (n = 17), schizoaffective disorder (SCA, n = 1), BD-I (n = 8) and acute psychotic episode F23 (n = 1). The wide phenotype of illness also included patients affected of mental disease, but who have not manifested any psychotic episode, as MDD (n = 14) and attention deficit hyperactive disorder (ADHD, n = 1). Within the narrow phenotype there were 10 females (24.3%) and 17 males (41.5%). By contrary, patients with a mental disease without psychosis included 12 females (29.3%) and only 3 males (7.3%). The mean age (± standard deviation) at participation was (30.5 ± 8) years for cases and (52 ± 6) years for controls.

### Linkage analysis identified a locus at 9q33.1–33.2 associated with psychiatric disorders (wide phenotype)

**Figure 2:**
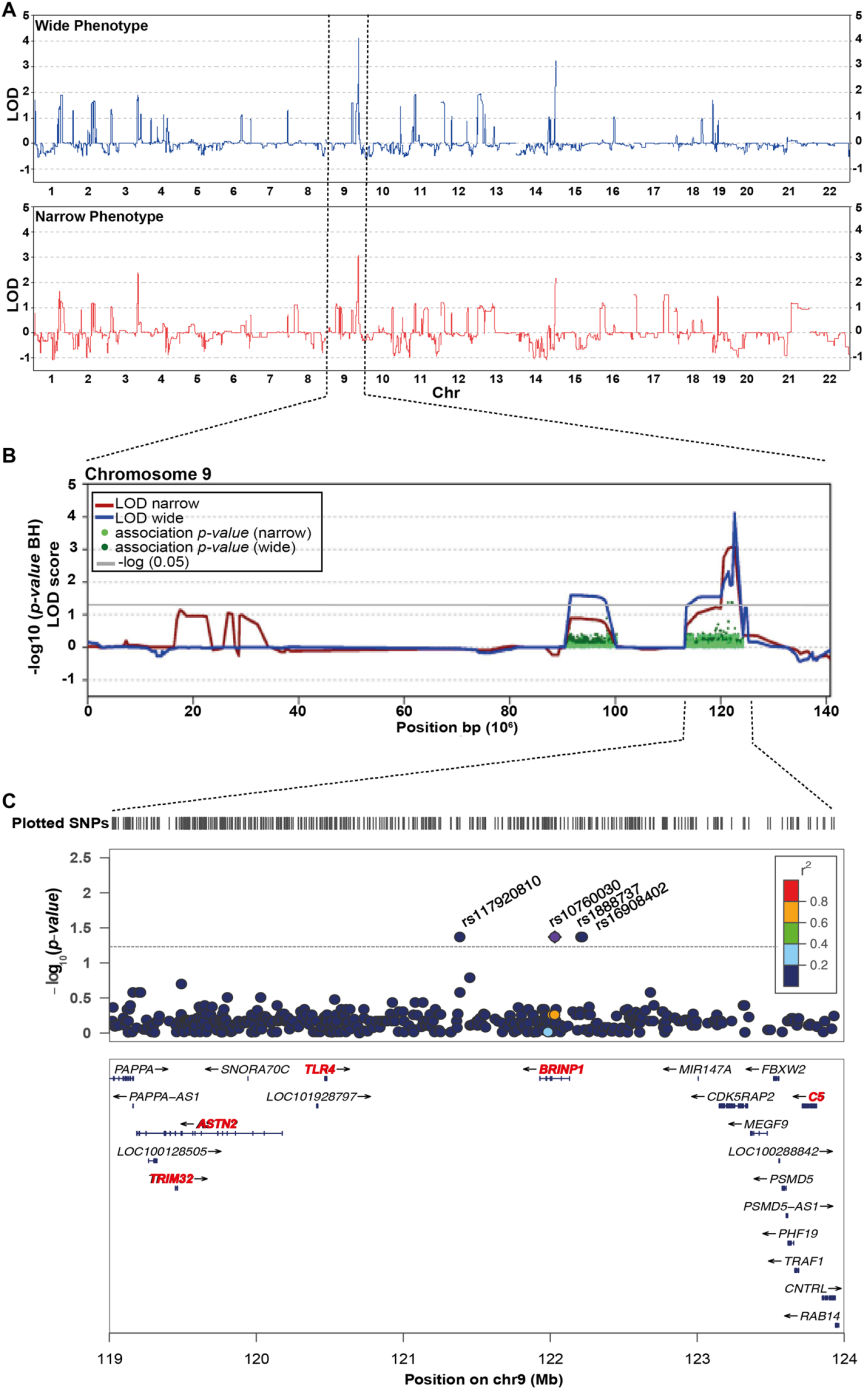
Genome-Wide Linkage Analysis identified the 9q33.1–33.2 linkage region. (**A**) Genome-wide results for the NPL score analysis. In blue represented the LOD scores for the wide phenotype; in red the LOD scores for the narrow phenotype. (**B**) NPL score results for Chromosome 9. The − log10 (*P* value) of the family-based association test in regions with significant NPL scores are shown as dark green or light green dots for the wide and narrow phenotype, respectively. (**C**) Regional association plot for the 9q33.1–33.2 linked region. The dashed grey line represents the significance threshold for the associated SNPs. In red, genes previously associated with MMD.

The genome-wide results for nonparametric LOD (NPL) scores for the wide and narrow phenotypes are plotted in Fig. 2A and Table 1. A genomic region on chromosome 9 (113,117,183–124,200,417; 11 Mb) highlighted with significant LOD scores (LOD wide = 4.11; LOD narrow = 3.07) (Fig. 2). Moreover, eight other genomic regions identified in both phenotype analyses reached LOD scores above 1.5 for the wide phenotype suggestive of linkage and were considered for further analyses (Fig. 2A and Table 1). Within those regions it is worth to mention the one at chromosome 3 (169,411,792–183,303,037; 13.89 Mb) with a LOD narrow = 2.36, and LOD wide = 1.89 (Table 1). Once linkage analysis was performed only considering the narrow phenotype, there were no linkage regions that reached significance, although ten regions had suggestive LOD scores ≥ 1, highlighting two regions of chromosome 17 with suggestive LOD scores of 1.5 (Chr17: 51,166–6,296,217, 6.24 Mb and Chr17: 33,006,378–35,752,691, 2.74 Mb) (Table S2A). Regarding the linkage analysis considering only the wide phenotype, no significant regions were identified, although thirteen regions had LOD scores ≥ 1 (Table S2B). Two regions at chromosome 19 had suggestive LOD scores > 1.5 (Chr19: 301,639–3,030,118, 2.72 Mb and Chr19: 5,892,954–7,900,562, 7.3 Mb) (Table S2B).

**Table 1:** Results of the NPL analysis and association analysis.

Linkage peaks					Significant SNPs wide association		Significant SNPs narrow association	
Chr	Start	End	LOD_max_ wide	LOD_max_ narrow	SNP	BH (*p*-value)	SNP	BH (*p*-value)
chr1	12,184,423	18,471,278	1.69	0.78	rs4661330	0.014146		
					rs6540592	0.02887		
chr1	203,605,690	226,875,552	1.89	1.64				
chr2	180,436,657	197,507,006	1.67	1.15	rs975417	0.031185		
chr3	16,656,331	36,251,715	1.35	1.03				
chr3	169,411,792	183,393,037	1.89	2.36				
chr9	90,405,210	100,270,886	1.59	0.9				
chr9	113,467,798	124,200,417	4.11	3.07	rs10760030	0.042617		
					rs117920810	0.042617		
					rs16908402	0.042617		
					rs1888737	0.042617		
chr11	24,669,006	29,134,515	1.91	1.14				
chr12	624,731	7,872,595	1.62	1.2				
chr13	20,957,230	32,355,432	1.93	1.06				

The coordinates within each chromosome (chr) are based on the human genome reference CRGh37/hg19.

*p*-values are adjusted by Benjamin–Hochberg (BH).

To narrow down the linkage regions of chromosomes 9 and 3, family-based association analyses were performed. 114 SNPs were found to be nominally significant on chromosome 9. Four SNPs (rs117920810, rs10760030, rs1888737 and rs16908402), associated with the wide phenotype analyses, remained significant after adjusting for multiple testing (*p*-value = 0.042617) (Fig. 2C, Table 1). The associated SNPs map at the highly conserved *ASTN2*/*BRINP1* locus at chr9q33.1–33.2, which contains five genes (*ASTN2*, *BRINP1*, *TRIM32*, *TLR4* and *C5*) that have been previously associated with neurodevelopmental disorders (reviewed in^29^). To closely analyze the linkage and the association region, haplotype estimation was conducted using SHAPEIT4^30^, phasing the entire chromosome 9 and carefully analyzing the linkage region (Figs. S2 and S3). Two different approaches were followed: we first used the SNP array genotyping to include all SNPs with MAF < 30% (Fig. S2). And second, haplotype phasing was also performed on those patients from whom we had whole genome sequencing data using SNPs with MAF < 0.5% (Fig. S3), searching for rare haplotypes that would segregate with the disease phenotypes. Only branch-specific haplotypes were identified. The subfamily 1 has three major haplotype blocks (H) shared by all affected subjects, but subject 1–2: H1 (chr9: 113,492,976–116,372,543, delimited by SNPs rs192009474–rs186260426, 2.87 Mb); H2 (chr9: 116,794,577–120,925,469, delimited by SNPs rs530450539–rs544077840, 4.1 Mb) and H3 (chr9: 121,694202–123,966,682, delimited by SNPs rs188485361–rs186909636, 2.2 Mb). It is important to emphasize that these three haplotypes are also shared by one healthy subject (1–21). The subfamily 3 has four haplotypes shared by wide affected subjects only: H1 (chr9: 116,857,705–117,615,594, delimited by SNPs rs34417627–rs142269627, 757 kb); H2 (chr9: 117,843,831–119,629,686, between SNPs rs944511–rs190965203, 1.78 Mb); H3 (chr9: 119,707,309–120,925,469, between SNPs rs118070509–rs544077840, 1.2 Mb); and H4 (chr9: 121,694,202–122,542,663, delimited by SNPs rs188485361–rs150164433, 848 kb), which is the haplotype located in the association region (Fig. S3).

On the other hand, on chromosome 3 a total number of 179 SNPs resulted to be significant, but none of them remained significant after correcting for multiple testing (Fig. S4). Family-based association analyses were also performed for all the suggestive linkage regions. Significant SNPs of common regions are summarized in Table 1, and those significant SNPs for the wide and narrow phenotype are summarized in Table S2.

Due to the high prevalence of psychiatric disorders in the pedigree, we hypothesized that related-affected subjects would share SNVs and structural variants (SVs) inherited from common ancestors within the linkage and the suggestive linkage regions identified.

### Identification of different CNVs in psychiatric and neurodevelopmental-associated loci

We next search for SVs in the linkage regions, first performing clinical karyotyping of four individuals (subjects 1–18, 1–25, 3–30 and 3–31) to discard structural variants, as balanced translocations. All four patients had normal karyotypes (data not shown). Next, we performed SNP-array based copy-number variant (CNV) analysis. Nine CNVs were identified (Table S3), but none of them were in the linkage regions 9q33.1–33.2 and 3q26.32–26.33. Remarkably, two psychotic patients (mother 3–11 and her son 3–31) harbored a 450 Kb duplication in the 3q29-schizophrenia locus^31,32^. This duplication shares 74.5% overlap with the 3q29 duplication syndrome, which is characterized by delayed development (particularly speech delay) and intellectual disability or learning difficulties, although its manifestation varies widely (DECIPHER and^31^). Moreover, the same subjects (3–11 and 3–31) plus the brother (3–12) of 3–11 also harbored a 127 Kb duplication at 4q35.2, a genomic region also associated with behavioral disorders as autism and ADHD (DECIPHER). Another interesting CNV identified was a 198 Kb duplication at 22q11.23, right next to the major risk locus for SCZ^33^. Phenotypes associated with duplications of similar size comprise cognitive impairment, emotional/affect behavior, hyperactivity and intellectual disability (DECIPHER and^34,35^). The mother 1–1, affected of MDD, transmitted the DUP22q11.23 to her two psychosis-affected children (1–18 and 1–19). Three other MDD subjects (3–9, 3–10 and 4–15) and a healthy control (3–34) also harbor the DUP22q11.23. It is also worth mentioning the deletion DEL12q14.1, only identified in affected subjects, that encodes the leucine rich repeats and immunoglobulin likes domain 3 (*LRIG3*) gene. Siblings 1–24, 1–27 (MDD) and 1–25 (SCZ) inherited this deletion from their mother 1–6 (MDD). Phenotypes associated with similar deletions at 12q14.1 include intellectual disability and delayed speech and language development (DECIPHER and^36^).

### SNVs and INDELs identified only in MMD subjects at 9q33.1–33.2

To search for rare (MAF < 0.01) coding SNVs and SVs below detection thresholds for SNP arrays, we conducted WGS (30× coverage) of 12 subjects: 8 affected of psychosis (1–2, 1–6, 1–18, 1–25, 3–11, 3–12, 3–30, 3–31), 2 MDDs (1–3, 3–13), and 2 healthy controls (1–21, 2–28). The genomic linkage region 9q33.1–33.2 and its surroundings were deeply analyzed. The coordinates used for variants identification were (chr9: 111,617,397–140,033,609). We first searched for rare SNVs with protein impact affecting conserved residues within 9q33.1–33.2. We did not identify any coding variant shared by all affected subjects within these coordinates. Six variants were identified in some affected subjects and were not present in any healthy control: two in *ZNF618* gene (Zinc Finger Protein 618, rs762985449 and rs770522574), one in *TNC* (Tenascin C, rs61729478), one in *CDK5RAP2* (CDK5 Regulatory Subunit Associated Protein 2, rs41296081), and two in *C5* (Complement C5, rs139479771 and rs34552775) (Table 2). Many other rare intronic or intergenic variants were only identified in affected subjects in the linkage chromosome 9 region (Table 2). Some of these rare SNVs were branch-specific and defined the four rare-haplotype blocks identified in the linkage region of Subfamily 3 (Table S5). In the association region, four rare intergenic variants (rs191347609, rs181505483, rs191352043, and rs4837653) are located within the H4 haplotype block of Subfamily 3 at chr9: 121,694,202–122,542,663 (Fig. S2 and Table S5), and they were shared by all the wide-affected individuals of subfamily 3.

**Table 2:** Rare genomic SNVs, INDELs and CNVs identified in the Chromosome 9 (chr9: 12,400,417–113,467,798) linked region.

	Gene	rs	bp	Ref	Obs	Protein impact	1000G	ExAC	gnomAD	Varsome	Subjects
**Rare SNPs coding regions**											
	ZNF618	rs762985449	116,811,096	G	A	R505H	NA	0.0000	0.0000	VUS	1–3, 1–6
		rs770522574	116,811,450	C	T	T623M	NA	0.0000	0.0000	VUS	1–18
	TNC	rs61729478	117,848,368	C	T	V548M	0.0064	0.0099	0.0091	Likely benign	3–31
	CDK5RA﻿P2	rs41296081	123,239,643	A	G	L571P	0.0032	0.0087	0.0084	Benign	1–6, 3–11, 3–12
	C5	rs139479771	123,762,323	C	A	A857S	0.0022	0.0000	0.0061	Likely benign	3–13, 3–30
		rs34552775	123,785,738	G	T	L360M	0.0010	0.0052	0.0053	Benign	1–6, 3–11, 3–12
	GSN	rs116185403	124,083,642	C	T	A481C	0.0016	0.0016	0.0030	Likely benign	1–2, 1–3, 1–6, 1–18, 1–21, 1–25
**Rare SNPs non-coding regions**											
Narrow	MUSK	rs149296909	113,479,998	T	C	Intronic	0.0058	NA	0.0030	Likely benign	3–11, 3–12, 3–30, 3–31
		rs368429456	113,481,468	G	T	Intronic	0.0058	NA	0.0028	Likely benign	3–11, 3–12, 3–30, 3–31
		rs183670470	113,515,498	G	A	Intronic	0.0012	NA	0.0018	VUS	3–11, 3–12, 3–30, 3–31
		rs79303192	113,525,126	G	A	Intronic	0.0014	NA	0.0020	VUS	3–11, 3–12, 3–30, 3–31
		rs149348343	113,548,471	G	A	Intronic	0.0010	NA	0.0019	VUS	3–11, 3–12, 3–30, 3–31
	LPAR1	rs146624388	113,642,528	G	T	Intronic	0.0010	NA	0.0012	VUS	3–11, 3–12, 3–30, 3–31
		rs147609101	113,681,896	G	A	Intronic	0.0070	NA	0.0065	VUS	3–11, 3–12, 3–30, 3–31
		rs72748167	113,684,505	G	A	Intronic	0.0038	NA	0.0071	VUS	3–11, 3–12, 3–30, 3–31
		rs375075858	113,727,420	T	C	Intronic	0.0008	NA	0.0014	VUS	3–11, 3–12, 3–30, 3–31
		rs187952001	113,727,561	G	T	Intronic	0.0008	NA	0.0015	VUS	3–11, 3–12, 3–30, 3–31
		rs72750194	113,745,730	A	C	Intronic	0.0030	NA	0.0074	VUS	3–11, 3–12, 3–30, 3–31
	NA	rs369362681	113,878,682	A	G	Intergenic	NA	NA	0.0049	VUS	3–11, 3–12, 3–30, 3–31
	C9orf84	NA	114,553,050	A	T	Intronic	Not reported			VUS	3–11, 3–12, 3–30, 3–31
	UGCG	rs182375662	114,669,858	G	A	Intronic	0.0014	NA	0.0014	VUS	3–11, 3–12, 3–30, 3–31
	NA	rs117263726	114,768,926	A	G	Intergenic	0.0020	NA	0.0020	VUS	3–11, 3–12, 3–30, 3–31
	PTBP3	rs147275436	115,011,769	G	A	Intronic	NA	NA	0.0001	VUS	3–11, 3–12, 3–30, 3–31
	KIAA1958	rs564854273	115,257,089	T	C	Intronic	0.0002	NA	0.0001	VUS	3–11, 3–12, 3–30, 3–31
		rs556349267	115,259,293	T	C	Intronic	0.0002	NA	0.0001	VUS	3–11, 3–12, 3–30, 3–31
		rs183650808	115,300,353	G	A	Intronic	0.0018	NA	0.0004	VUS	3–11, 3–12, 3–30, 3–31
		rs144366338	115,303,659	T	C	Intronic	0.0014	NA	0.0014	VUS	3–11, 3–12, 3–30, 3–31
	NA	rs574327747	115,443,505	T	A	Intergenic	0.0020	NA	0.0005	VUS	3–11, 3–12, 3–30, 3–31
	FKBP15	rs141811808	115,960,045	A	G	Intronic	0.0040	NA	0.0023	Likely benign	3–11, 3–12, 3–30, 3–31
		NA	115,978,421	A	G	Intronic	NA	NA	0.0017	VUS	3–11, 3–12, 3–30, 3–31
	NA	rs151159543	116,144,471	T	C	Intergenic	0.0058	NA	0.0096	VUS	3–11, 3–12, 3–30, 3–31
	ZNF618	rs530450539	116,794,577	A	C	Intronic	0.0008	NA	0.0020	VUS	3–11, 3–12, 3–30, 3–31
	DELEC1	rs537057975	118,135,400	A	G	Intronic	0.0002	NA	0.0000	VUS	3–11, 3–12, 3–30, 3–31
Wide	NA	rs145813581	116,883,714	A	G	Intergenic	0.0036	NA	0.0077	VUS	3–11, 3–12, 3–13, 3–30, 3–31
	COL27A1	rs187396762	116,999,043	G	T	Intronic	0.0020	NA	0.0061	VUS	3–11, 3–12, 3–13, 3–30, 3–31
		rs117732536	117,017,282	G	A	Intronic	0.0046	NA	0.0085	Likely benign	3–11, 3–12, 3–13, 3–30, 3–31
	WHRN	NA	117,180,316	A	G	Intronic	Not reported			VUS	3–11, 3–12, 3–13, 3–30, 3–31
	NA	rs72754560	117,491,583	A	G	Intergenic	0.0048	NA	0.0061	VUS	3–11, 3–12, 3–13, 3–30, 3–31
	NA	rs146845932	117,503,577	C	T	Intergenic	0.0056	NA	0.0062	VUS	3–11, 3–12, 3–13, 3–30, 3–31
	NA	rs56000964	117,523,825	C	T	Intergenic	0.0048	NA	0.0061	VUS	3–11, 3–12, 3–13, 3–30, 3–31
	NA	rs56008628	117,531,509	C	T	Intergenic	0.0034	NA	0.0050	VUS	3–11, 3–12, 3–13, 3–30, 3–31
	NA	NA	117,754,194	C	A	Intergenic	NA	NA	0.0000	VUS	3–11, 3–12, 3–13, 3–30, 3–31
	TNC	rs944511	117,843,831	G	T	Intronic	0.0020	NA	0.0030	VUS	3–11, 3–12, 3–13, 3–30, 3–31
		rs2482079	117,868,293	T	C	Intronic	0.0022	NA	0.0030	VUS	3–11, 3–12, 3–13, 3–30, 3–31
		NA	117,878,534	G	A	Intronic	NA	NA	0.0000	VUS	3–11, 3–12, 3–13, 3–30, 3–31
	DELEC1	rs2992149	117,926,298	G	A	Intronic	0.0040	NA	0.0035	VUS	3–11, 3–12, 3–13, 3–30, 3–31
		NA	118,116,520	A	G	Intronic	NA	NA	0.0000	VUS	3–11, 3–12, 3–13, 3–30, 3–31
		rs537057975	118,135,400	A	G	Intronic	0.0002	NA	0.0000	VUS	3–11, 3–12, 3–13, 3–30, 3–31
		rs545046369	118,142,492	G	A	Intronic	0.0002	NA	0.0001	VUS	3–11, 3–12, 3–13, 3–30, 3–31
	NA	rs571168692	118,405,585	C	T	Intergenic	0.0002	NA	0.0000	VUS	3–11, 3–12, 3–13, 3–30, 3–31
	NA	NA	118,522,840	G	A	Intergenic	NA	NA	0.0000	VUS	3–11, 3–12, 3–13, 3–30, 3–31
	NA	NA	118,550,150	C	T	Intergenic	NA	NA	0.0000	VUS	3–11, 3–12, 3–13, 3–30, 3–31
	NA	rs146826488	118,613,045	A	G	Intergenic	0.0094	NA	0.0040	VUS	3–11, 3–12, 3–13, 3–30, 3–31
	NA	rs147559462	118,858,914	A	G	Intergenic	0.0032	NA	0.0040	VUS	3–11, 3–12, 3–13, 3–30, 3–31
	PAPPA	NA	118,976,797	C	A	Intronic	Not reported			VUS	3–11, 3–12, 3–13, 3–30, 3–31
		**NA**	**119,022,095**	**T**	**C**	**Intronic**	**Not reported**			**VUS**	**3–11, 3–12, 3–13, 3–30, 3–31**
		**NA**	**119,024,387**	**G**	**A**	**Intronic**	**Not reported**			**VUS**	**3–11, 3–12, 3–13, 3–30, 3–31**
		**rs181003102**	**119,063,932**	**A**	**T**	**Intronic**	**0.0010**	**NA**	**0.0036**	**VUS**	**3–11, 3–12, 3–13, 3–30, 3–31**
	**ASTN2**	**rs186435615**	**119,198,874**	**G**	**A**	**Intronic**	**0.0008**	**NA**	**0.0033**	**VUS**	**3–11, 3–12, 3–13, 3–30, 3–31**
		**rs139814838**	**119,219,573**	**A**	**G**	**Intronic**	**0.0028**	**NA**	**0.0080**	**Likely benign**	**3–11, 3–12, 3–13, 3–30, 3–31**
		**NA**	**119,270,176**	**T**	**C**	**Intronic**	**Not reported**			**VUS**	**3–11, 3–12, 3–13, 3–30, 3–31**
		**rs116995922**	**119,318,987**	**C**	**T**	**Intronic**	**0.0028**	**NA**	**0.0077**	**VUS**	**3–11, 3–12, 3–13, 3–30, 3–31**
		**rs117517893**	**119,325,920**	**C**	**T**	**Intronic**	**0.0030**	**NA**	**0.0083**	**VUS**	**3–11, 3–12, 3–13, 3–30, 3–31**
		**rs188489544**	**119,340,790**	**T**	**C**	**Intronic**	**0.0028**	**NA**	**0.0077**	**VUS**	**3–11, 3–12, 3–13, 3–30, 3–31**
		**rs150169463**	**119,371,830**	**T**	**C**	**Intronic**	**0.0028**	**NA**	**0.0078**	**VUS**	**3–11, 3–12, 3–13, 3–30, 3–31**
		**NA**	**119,437,954**	**G**	**A**	**Intronic**	**Not reported**			**VUS**	**3–11, 3–12, 3–13, 3–30, 3–31**
		**rs117579154**	**119,484,619**	**A**	**G**	**Intronic**	**0.0026**	**NA**	**0.0088**	**VUS**	**3–11, 3–12, 3–13, 3–30, 3–31**
		**rs534176897**	**119,684,901**	**C**	**T**	**Intronic**	**NA**	**NA**	**0.0000**	**VUS**	**3–11, 3–12, 3–13, 3–30, 3–31**
		**rs186219478**	**119,816,588**	**T**	**C**	**Intronic**	**0.0016**	**NA**	**0.0048**	**VUS**	**3–11, 3–12, 3–13, 3–30, 3–31**
		**rs117473036**	**119,961,964**	**T**	**C**	**Intronic**	**0.0020**	**NA**	**0.0048**	**VUS**	**3–11, 3–12, 3–13, 3–30, 3–31**
		**rs146129313**	**119,966,382**	**G**	**C**	**Intronic**	**0.0016**	**NA**	**0.0040**	**VUS**	**3–11, 3–12, 3–13, 3–30, 3–31**
		**rs149483580**	**120,026,166**	**C**	**T**	**Intronic**	**0.0016**	**NA**	**0.0052**	**VUS**	**3–11, 3–12, 3–13, 3–30, 3–31**
		**rs191594200**	**120,105,136**	**G**	**A**	**Intronic**	**0.0004**	**NA**	**0.0011**	**VUS**	**3–11, 3–12, 3–13, 3–30, 3–31**
	**NA**	**rs191347609**	**121,565,707**	**G**	**A**	**Intergenic**	**0.0004**	**NA**	**0.0029**	**VUS**	**3–11, 3–12, 3–13, 3–30, 3–31**
	**NA**	**rs117766598**	**121,868,452**	**T**	**C**	**Intergenic**	**0.0032**	**NA**	**0.0080**	**VUS**	**3–11, 3–12, 3–13, 3–30, 3–31**
	**NA**	**rs181505483**	**122,175,429**	**A**	**G**	**Intergenic**	**0.0022**	**NA**	**0.0018**	**VUS**	**3–11, 3–12, 3–13, 3–30, 3–31**
	**NA**	**rs191352043**	**122,192,299**	**T**	**C**	**Intergenic**	**0.0020**	**NA**	**0.0015**	**VUS**	**3–11, 3–12, 3–13, 3–30, 3–31**
	**NA**	**rs4837653**	**122,292,842**	**A**	**G**	**Intergenic**	**0.0006**	**NA**	**0.0008**	**VUS**	**3–11, 3–12, 3–13, 3–30, 3–31**
	**NA**	**rs186447793**	**122,530,344**	**G**	**A**	**Intergenic**	**0.0022**	**NA**	**0.0011**	**VUS**	**3–11, 3–12, 3–13, 3–30, 3–31**
	**NA**	**rs150164433**	**122,542,663**	**A**	**G**	**Intergenic**	**0.0030**	**NA**	**0.0091**	**VUS**	**3–11, 3–12, 3–13, 3–30, 3–31**
	**NA**	**rs191966830**	**122,555,982**	**T**	**G**	**Intergenic**	**0.0054**	**NA**	**0.0032**	**VUS**	**3–11, 3–12, 3–13, 3–30, 3–31**
	**NA**	**rs145380362**	**122,611,729**	**T**	**C**	**Intergenic**	**0.0062**	**NA**	**0.0045**	**VUS**	**3–11, 3–12, 3–13, 3–30, 3–31**
**Rare INDELS non-coding regions**											
	DELEC1	NA	118,113,219	AAGG	A	Intronic	NA	NA	0.0001	VUS	3–11, 3–12, 3–13, 3–30, 3–31
	LINC00474	NA	118,667,077	ATG	A	Intronic	NA	NA	0.0001	VUS	3–11, 3–12, 3–13, 3–30, 3–31
	HSDL2	NA	115,184,679	TCCTGACATAAG	T	Intronic	NA	NA	NA	VUS	3–9, 3–10, 3–11, 3–12, 3–30, 3–31, 3–33
	ZNF618	NA	116,646,033	A	ATAT	Intronic	NA	NA	NA	VUS	3–8, 3–30, 3–34
	DELEC1	NA	118,111,076	TCA	T	Intronic	NA	NA	NA	VUS	3–30
	**PAPPA**	**rs748913197**	**119,154,431**	**G**	**GCACA**	**Intronic**	**NA**	**NA**	**NA**	**VUS**	**1–24, 1–25, 1–26, 1–27**
	**ASTN2**	**NA**	**120,127,502**	**GAA**	**G**	**Intronic**	**NA**	**NA**	**NA**	**VUS**	**1–25**

	Gene	Start	End	CNV	Size (bp)	Protein impact	Decipher				
**CNVs coding regions**											
	DELEC1	118,067,401	118,070,500	DEL	3099	Gene	Not reported				1–18, 1–19
**CNVs non-coding regions**											
	NA	113,669,166	113,669,260	DEL	94	inton_variant	Not reported				1–18, 1–19, 3–13
	NA	114,597,069	114,597,147	DEL	78	Intergenic	Not reported				1–2, 1–5
	NA	116,463,148	116,463,353	DEL	205	Intergenic	Not reported				3–29, 3–31, 3–33
	**NA**	**120,596,847**	**120,597,027**	**DEL**	**180**	**Intergenic**	**Not reported**				**1–18, 1–19, 3–8, 3–30**

In bold are highlighted the genomic rare variants identified in the associated chr9q33.1–33.2 coordinates.

VUS variant of uncertain significance, NA not available.

We next searched for small SVs that could not be detected by SNP-array, using different algorithms, HaplotypeCaller of GATK, CNVnator, Manta, BreakDancerMax and CREST. CNVnator identified 7 non-reported small INDELs in non-coding regions, only in MMD subjects, affecting the genes *LPAR1*, *HSDL2*, *DELEC1*, *PAPPA*, *ASTN2* and *ZNF618* (Table 2). Two of these INDELs, located at the H2 haplotype of subfamily 3 (Fig. S2 and Table S5), were shared by all the MMD subjects of the subfamily 3 and were not present in the healthy controls: a three base pair (AGG) deletion in an intronic region of *DELEC1* gene (chr9: 118,113,219), predicted to affect a histone H3 lysine 4 trimethylation (H3K4me3) site in the brain frontal cortex^37^, and a two base pairs (TG) deletion at the long intergenic non-coding RNA 474 (*LINC00474*) (chr9: 118,667,077) (Table 2 and Table S5)). Moreover, CNVnator also identified a non-reported larger deletion of 3099 bp that overlapped with the expression of *DELEC1* gene (Table 2). This deletion is only present in two psychotic siblings, 1–18 (SCZ) and 1–19 (BD-I). Manta identified four other non-reported deletions in intergenic regions at 9q33.1–33.2 (Table 2). All these INDELs were checked by PCR and Sanger sequencing and were not identified in any healthy family control.

The search for rare SNVs was extended to the rest of the genome. In Supplementary Table S4 are summarized the coding rare SNVs identified in susceptible linkage regions. Only the rs145032100 in the *ARHGAP19* gene was shared by all affected subjects but was also carried by some healthy controls (Table S4A). This SNV is located at chr10q24.1, a suggestive region associated with the narrow phenotype (LOD score = 1.02).

### Regions associated with the wide phenotype are enriched for genes involved in voltage-gated ion channels, microtubule organization and immune system

**Figure 3:**
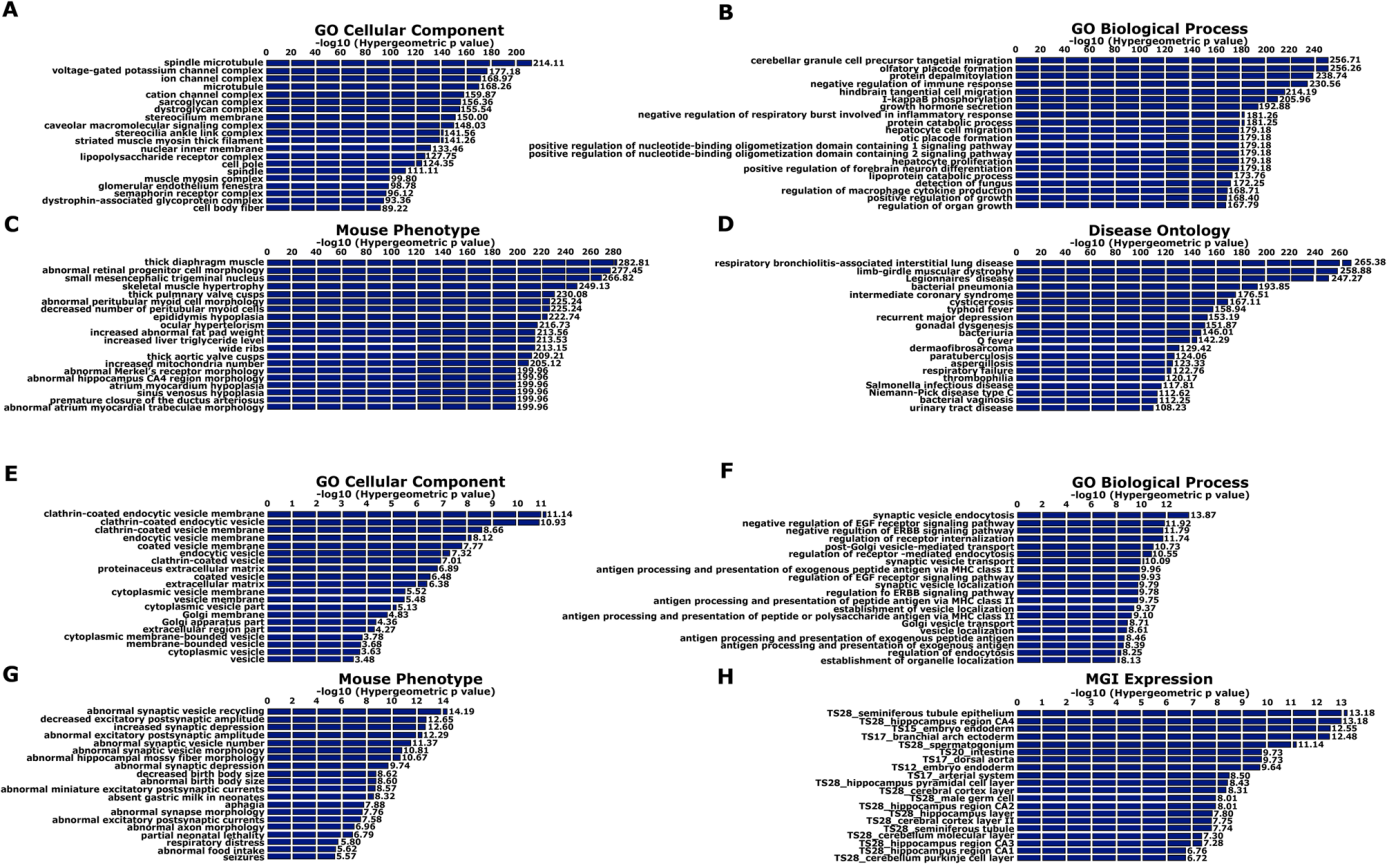
MMD is enriched for genes associated with the immune system and the cytoskeleton of tubulin (**A**–**D**). Psychosis is enriched for genes involved in synaptic function (**E**–**H**). GO term enrichment analyses with GREAT^38^, including the significant SNPs of the suggestive linkage regions with LOD > 1.5 for the wide phenotype (**A**–**D**) and with LOD > 1 for the narrow phenotype (**E**–**H**). GO terms identified by: (**A**, **E**) Molecular Function; (**B**, **F**) Biological Process; (**C**, **G**) Cellular Component; and (**D**, **H**) Disease Ontology.

Functional enrichments were performed using GREAT^38^, searching for gene ontology (GO) terms associated with the significant SNPs of the linked region 9q33.1–33.2 plus the ones identified by both phenotype analyses (LOD > 1.5) (Table 1). The background used was composed of all the filtered SNPs, previously used to perform the association analysis. Regarding GO cellular component highlighted ontologies associated with voltage-gated ion channels and tubulin cytoskeleton (Fig. 3A). Interestingly, GO Biological Process terms were enriched for genes related to neuronal migration and differentiation and genes associated with the immune response (Fig. 3B). Mouse Genome Informatics (MGI) identified enriched expression in cerebral cortex (Fig. 3C), and within GO Disease Ontology, recurrent major depression appeared as the eight most significant enriched term (Fig. 3D).

### Regions associated with the narrow phenotype are enriched for genes involved in synaptic vesicle function

We also investigated whether the significant SNPs associated with the narrow phenotype from the suggestive narrow linkage regions (LOD > 1) (Table 1 and Table S2A) showed functional enrichments related to the disease etiology. Interestingly those regions appeared enriched for synaptic vesicle function, composition and transport (Fig. 3E–H).

### Psychotic subjects have increased risk associated with common variants

We finally measured polygenic risk scores (PRS) to evaluate the contribution of common variants to the psychotic phenotype. PRS were calculated using GWAS data from^9^. We observed a clear gradient in the PRS results. All psychotic members of the pedigree scored positive PRS, either using SCZ as a base dataset to calculate the PRS or the combination of SCZ and BD (Table S6). By contrary, some subjects affected of MDD had negative PRS scores (subjects 1–24, 1–22, 4–15 and 1–20), suggestive of being protective. Interestingly, out of the 13 healthy controls analyzed, only one subject scored positive PRS (4–14, PRS = 0.36) (Table S6).

## Discussion

The genetics paradigm of mental illness has changed substantially in recent years. Families with high prevalence, such as the one studied, are expected to encode variants of large effect. But since MMDs are polygenic, we obviously cannot search for a single cause of the disease and whole genome approaches need to be made.

In this pedigree we identified a susceptibility locus with a predominant involvement, the 9q33.1–33.2. Previous linkage analysis in families with mental disorders have reported the same region or very close coordinates, some of which could be considered partial linkage replications^39–41^. Badenhop et al. found suggestive evidence for linkage for 9q31–q33 when analyzing 13 families with high prevalence of BD-I^39^. Kaufmann et al*.* found suggestive evidence for linkage for 9q32–9q34 when analyzing 30 nuclear SCZ African–American families comprising 98 subjects (NPL Z_max_ = 2.17, p = 0.017)^40^. Interestingly, the highest evidence for linkage was found when considering individuals diagnosed with either BD I and II, SZA manic type, or depression as affected (NPL = 2.5 between D9S1690 and D9S1677 at 9q31–q33)^40^. Venken et al*.* found evidence of linkage at 9q31.1–q33 for affective disorder susceptibility analyzing nine multigenerational families from Northern Sweden^41^. Interestingly, some of these linkage reports have included depression and BD in their analyses; this is congruent with our report of linkage which is higher when including depressive subjects as affected (wide phenotype). Others have reported linkage peaks very close to the one identified in here^42–44^. Labbe et al. found suggestive evidence for linkage at 9q33 adopting a symptom dimension approach for delusional symptoms, in where most of the patients contributing to this signal were diagnosed as SCZ^42^. Liu et al*.* found suggestive evidence for linkage at 9q31 analyzing 373 individuals from 40 BD pedigrees^43^. Park et al*.* also found evidence for linkage at 9q31 analyzing psychotic BD in 40 extended pedigrees comprising 373 individuals^44^. Interestingly, the same genomic region 9q33.1–33.2 has also been associated with psychotic disorders through GWAS^45^.

The linked 9q33.1–33.2 region contain five candidate genes from the immune system that participate in synaptic processes and have been previously associated with neurodevelopmental disorders, *ASTN2*, *BRINP1*, *C5*, *TLR4* and *TRIM32*. *ASTN2* (Astrotactine 2) and *BRINP1* (Bone Morphogenetic Protein/Retinoic Acid-inducible Neural specific Protein) encode proteins from the Membrane Attack Complex Perforin (MACPF) family, highly expressed in the developing brain (reviewed in^29^). Both genes have been associated with SCZ^45,46^, BD-I^47^, and other neurodevelopmental disorders^48^ and with structural abnormalities of the hippocampal volume^49^. ASTN2 facilitates glial-guided migration during brain development^50^, and regulates synaptic trafficking by modulating the composition of surface synaptic vesicle proteins^51^. BRINP1 function in neuronal development is less studied, although it has been implicated in neurogenesis^52^ and cell cycle regulation^53^. In fact, *Brinp1* knock-out (*KO*) mice evidence altered hippocampal neurogenesis^52^ and exhibit altered behaviors that could model MMDs^52,54^.

*C5*, which encodes Complement 5 protein, is another interesting candidate gene in the linked region. Recent evidences has implicated the complement system as a promising immune mediator of SCZ (reviewed in^55^). GWAS studies have identified association of complement components as *C4* and *CSMD1* with SCZ^8,56^. In addition to the genetic findings, different studies have reported increased complement expression and overall activity in the plasma or serum of SCZ patients (reviewed in^55^). Recently, increased C5 levels have also been observed in cerebrospinal fluid of SCZ patients^57^.

*TLR4* encodes the Toll-like Receptor 4, which plays a fundamental role in pathogen recognition and activation of innate immunity. TLRs express in the developing and adult CNS, in where have been involved in neurogenesis, axonal growth and structural plasticity (reviewed in^58^). Altered TLR4 counts have been observed in SCZ patients (reviewed in^59^), and interestingly antipsychotic treatment could normalize those counts^60^. Increased TLR4 expression has also reported in postmortem frontal cortex from SCZ patients and depressed suicide victims (reviewed in^59^). Other evidence supporting the role of TLR4 on psychiatric diseases come from animal models. TLR4 *KO* mice show improved spatial memory^61^, due to increased neuronal progenitor cell proliferation and neuronal differentiation in the hippocampus, suggesting that TLR4 may act to reduce hippocampal neurogenesis^62^.

The last candidate gene in the linked region is *TRIM32,* a small gene nested within an intron of *ASTN2* and transcribed from the opposite strand. It encodes the Tripartite motif‐containing protein 32 (TRIM32). TRIM32 is a cell fate-determinant for a balanced embryonic development of the neocortex^63^ and the adult neurogenesis^64^. Recent reports have associated *TRIM32* with psychiatric disorders, such as MDD, ASD, ADHD, anxiety and obsessive–compulsive disorder (reviewed in^48^). Interestingly, *TRIM32* loss protects against the development of anxiety and depression induced by chronic stress^65^.

Although we did not find any coding variant in the linked region that segregates with all affected MMD subjects, we found several rare SNVs in those genes harbored only by MMD patients. Moreover, we cannot discard a regulatory role of the genomic region containing the two small INDELs identified at *DELEC1 (deleted in esophageal cancer 1)* gene and at *LINC00474* in all affected subjects of subfamily 3. Further studies will have to shed light on the potential pathogenic roles of the INDELs and the rare SNVs identified in the linked region.

Furthermore, it is also interesting to highlight the only coding rare SNV identified in the family which segregates with all affected subjects, the g.99006061 G>A transition at *ARHGAP19,* associated with the psychotic phenotype. ARHGAP19 is another hematopoietic cell regulator, a specific Rho GTPase-activating protein (GAP) that plays an essential role in the division of T lymphocytes^66^.

Overall, our results reinforce the growing evidence linking immune system modulators with specific brain functions and MMDs. The susceptibility locus 9q33.1–33.2 should be taken into consideration in further genetic analysis, especially in those families that come from the same region.

## Methods

### Clinical assessments

Psychiatric assessments included semi-structured interviews, using the Spanish version of the Structured Clinical Interview for Diagnostic and Statistical Manual of Mental Disorders (DSM-IV) Axis I (SCID-I)^67^, GAF^68^, PANSS^69^ and the Diagnostic Interview for Genetic Studies (DIGS)^70^.

### Sample collection

A total of n = 34 subjects were recruited, DNA samples were obtained, being n = 9 psychotic patients, n = 11 non-psychotic mental disorder patients and n = 14 healthy controls. Genomic assays were done on n = 34 individuals, including SNP arrays (n = 34), WGS (n = 12), and karyotyping (n = 4).

### Ethics

The experimental protocol was approved by the ethics committee of the Balearic Islands (CEI-IB) and was carried out in accordance with the ethical standards of the 2013 Declaration of Helsinki. All studied family members gave their written informed consent to take part in the study.

### Genotype data

#### Genotyping and SNP array

Whole-genome genotype was generated for all samples in the Research Unit of Molecular Epidemiology, Institute of Epidemiology II, Helmholtz Zentrum München, German Research Center for Environmental Health using the Infinium Global Screening Array-24 v1.0 (GSA) from Illumina, which includes 642,824 SNPs. In addition, a pool of 57,254 SNPs (Multi-disease Drop-In Panel (MD)) previously related to neurological disorders was also genotyped. The genotype calling and CNV analysis were performed using the Genome Studio 2.0 (Illumina Inc. San Diego, California, USA).

Nonparametric linkage (NPL) analysis was carried using the NPL scoring function^71^, implemented in Merlin v1.1.2^72^. Evidence for Linkage was assessed with the Kong and Cox exponential model^73^. Allele frequencies were calculated using the maximum likelihood method. Due to the complexity of the pedigree, it was split up in three different ~ 24 bit-sized sub-pedigrees (See Fig. 1). Before running linkage, data was exhaustively quality controlled. Graphical Representation of Relationship Errors (GRR)^74^ was used to identify errors in the structure of the pedigree. Whole Genome Association Analysis Toolset (PLINK 1.7)^75^ was used for the SNPs quality control. SNPs were excluded when Minor Allele Frequency (MAF) < 0.05, and if showing Mendelian inconsistencies. A total of 1198 Mendelian inconsistencies were found (0.17%). Unlikely double recombinants were analyzed using the “error detection” option from Merlin v1.1.2 and subsequently excluded using the “pedwipe” option. Linkage was carried using the most heterozygous SNPs per chromosome after being modeled for LD. Model for LD was performed calculating r^2^ using PLINK and removing one SNP of a pair each time r^2^ > 0.5. Out of the initial 700,008 SNPs genotyped, 8,078 SNPs were selected for the analysis.

#### Association analyses of suggestive linkage regions and haplotyping

Family-based association analyses were conducted using the Linkage and Association Modelling in Pedigrees Software (LAMP)^76^. We included all the SNPs from significant and suggestive linkage regions, using both definitions of the phenotype, wide and narrow. The *p*-values were corrected for multiple testing using the Benjamini–Hochberg correction. LAMP allows to accommodate different family structures.

Haplotype phase was estimated using SHAPEIT 4 (version 4.2)^30^ and haplotypes were visualized using inPHAP^77^ mapping SNPs shared by at least four affected subjects. To perform phasing two approaches were followed: (1) Genotyped SNPs from SNP array with low minor allele frequency (MAF ≤ 30%) were included. (2) Phasing was also performed using SNPs with MAF < 1% from VCF files of WGS. Allele frequencies were extracted from gnomAD. For both approaches, SNPs with high individual missingness rate (> 80%), and high genotyping missingness rate (> 80%) were excluded. SNPs that were not called in all the genotyped subjects were also excluded. Chromosomes 9 was entirely phased.

#### Whole genome sequencing (WGS)

12 samples were whole-genome sequenced (1–2, 1–3, 1–5, 1–18, 1–21, 1–25, 3–11, 3–12, 3–13, 3–30, 3–31, 2–28) using the BGISEQ-500 service (BGI Genomics Co., Ltd.). The workflow to obtain variant call format (VCF) files from raw data (FASTQ) provided by BGI was based on GATK Best Practices. FASTQ files, containing raw unmapped reads and Phred scores were quality controlled using FastQC tool. Low-quality sequences (phred score < 20) and adaptors were removed using cutadapt. QC sequences were aligned against the reference human genome (GRCh37/hg19) using BWA-MEM algorithm implemented in Burrows–Wheeler Alignment tool (BWA). Aligned data in SAM (Sequence Alignment/Map) format were then sorted and converted into BAM files using SAMtools. To generate new BAM files, PCR duplicates were removed using Picard Tools and realignment around INDELs and base recalibration was performed (BQRS) using Genome Analysis Toolkit (GATK). SNP and INDEL calling were carried from the cleaned BAM files using GATK producing unfiltered primary VCF files; which were then filtered using the variant call recalibration procedure (VSQR) to generate the definitive VCF files. VCF files were directly analyzed using ENLIS Genome Research V1.9 (Berkeley, CA, USA) which uses its own annotation pipeline. Shared variation among affected individuals was filtered for read depth > 10 and MAF < 0.01, using ENLIS Genome Research V1.9 (Berkeley, CA, USA). Alternatively, VCF files were annotated using SnepEff^78^ including the prediction of different protein impact and conservation algorithms and allele frequencies from 1000G (https://www.internationalgenome.org/) and gnomAD (gnomAD; https://gnomad.broadinstitute.org). The resulting txt files generated were analyzed for rare, shared variation among affected individuals using R.

### CNV and SVs detection

CNVs were analyzed from WGS and from SNP array data.

CNVs and SVs detection from WGS data were performed taking advantage of the paired-end sequencing configuration of the samples, and using the following algorithms: (1) CNVnator^79^, a read-depth based algorithm which is useful for detecting large INDELs, insertions and deletions; (2) BreakDancerMax^80^, a paired-read based algorithm, that allows the detection of large SVs such as deletions, insertions, inversions, and intrachromosomal and interchromosomal translocations; (3) CREST^81^, an split-read based algorithm that also allows the detection of the same SVs as BreakDancerMax; (4) Manta^82^, which combines both split-read and read-pair methods and it is useful for detecting large SVs, medium-sized INDELs and large insertions; and (5) HaplotypeCaller of GATK (v3.3.0), which was used for small INDELs detection (< 50 bp). CNVnator was run in all WGS samples using standard settings and a bin size of 100 bp (optimized for 20–30× coverage). Manta was run as a joint diploid sample analysis. BreakDancerMax and CREST were used with default settings.

CNVs were also detected from SNP array data using GenomeStudio 2.0 (Illumina Inc. San Diego, California, USA), taking as a reference GRCh37/hg19. This algorithm is based on two parameters: the B allele frequency (BAF) and the Log R Ratio (LRR) which can be used to test the genotyping quality of the samples and to check the presence of CNVs across the genome. The BAF is a measure of allelic imbalance. In a normal well-genotyped sample, three genotypes are expected, homozygous AA, heterozygous AB, and homozygous BB. Once referred to the B allele, BAF is expected to have three discrete values, 0, 0.5, and 1 (representing AA, AB, and BB genotypes, respectively). R is defined as the sum of the probe intensities used to genotype the different markers. When it is normalized becomes the LRR which is a measure of relative intensity, the logarithm (base 2) of the observed value of R (observed probe intensity) divided by the expected value (expected probe intensity)^83^.

All variants identified using the different algorithms were checked on the bam files using the software IGV, designed to visualize genomic data. This allowed the detection of artifacts or variants called in low coverage regions. Non-previously reported INDELs located on the linkage chromosome 9q33.1–33.2 were validated in all the studied subjects of the family by PCR followed by electrophoresis and Sanger sequencing.

#### Analysis of rare SNP and CNV variants

SIFT (http://sift.jcvi.org/)^84^, Polyphen (http://genetics.bwh.harvard.edu/pph2/)^85^, VarSome (http://varsome.com)^86^ and UniProt (https://www.uniprot.org)^87^ were used to predict the levels of variant penetrance. DisGeNET (http://www.disgenet.org/web/DisGeNET/menu)^88^, VarElect (http://varelect.genecards.org/), and Schizophrenia Exome Sequencing Genebook^89,90^ were also used to characterize variations. DECIPHER (DatabasE of genomiC varIation and Phenotype in Humans using Ensembl Resources; https://www.deciphergenomics.org)^91^ and CNVxplorer (http://cnvxplorer.com)^92^ were used to study the pathogenicity and conservation of the identified CNVs. All genomic data for molecular variants in this study were compatible with Genome build GRCh37. Database of genomic variants (DGV; http://dgv.tcag.ca/dgv/app/home)^93^ and Integrative Genomics Viewer (IGV; http://software.broadinstitute.org/software/igv/)^94^ were used for SNP analysis.

Functional enrichment of biological pathways was investigated using the online tool GREAT (Genomic Regions Enrichment of Annotations Tool; http://great.stanford.edu/public/html/)^38^. The enrichment analyses were based on the comparison between significant SNPs associated with the phenotype and the rest of the SNPs of the SNP array.

Polygenic risk scores (PRS) were calculated for each family member (n = 34) using the PRSice-2 v2.1.11, with the publicly available PGC schizophrenia GWAS as a base dataset (33,426 SCZ cases, 54,065 controls), in addition to BD (20,129 BD cases, 21,524 controls). Before computing PRS, data was quality controlled for missingness per SNP and per subject (excluding sample with a rate of missingness higher than 10%), assigned sex inconsistencies, MAF < 0.05 in the dataset, deviances from Hardy–Weinberg equilibrium and Mendelian inconsistencies. After quality control, data from the target dataset was transformed to match the base dataset. This step is vital since any inconsistencies in the effective allele (A1) might have a profound impact on the results. PRS were calculated with default clumping settings and normalizing PRS scores.

### Ethics approval

The present study was approved by the ethics committee of the Balearic Islands (CEI-IB), Spain.

### Consent to participate

Written informed consent was obtained from all studied family members.

## Supplementary Information

Supplementary Information.

## Data Availability

The datasets generated for this study can be shared upon reasonable request and are publicly available in the European Genome-Phenome Archive (EGA) (https://ega-archive.org/studies/EGAS00001004592).
