# Supplementary material for: The conserved ASTN2/BRINP1 locus at 9q33.1–33.2 is associated with major psychiatric disorders in a large pedigree from Southern Spain
Source: Sci Rep. 2021 Jul 15;11:14529. doi: 10.1038/s41598-021-93555-4 (PMC8282839; doi:10.1038/s41598-021-93555-4)
Supplement: Supplementary file 1 — Supplementary Information. [file 41598_2021_93555_MOESM1_ESM.docx]

**TITLE:** The conserved *ASTN2*/*BRINP1* locus at 9q33.1-33.2 is associated with major psychiatric disorders in a large pedigree from Southern Spain.

RUNING TITLE: 9q33.1-33.2 locus associated with psychosis

AUTHORS: Josep Pol-Fuster^1,2^, Francesca Cañellas^2,3^, Laura Ruiz-Guerra^2^, Aina Medina-Dols^2^, Bàrbara Bisbal Carrió^1,2^, Bernat Ortega-Vila^2,4^, Jaume Llinàs^1^, Jessica Hernandez-Rodriguez^2^, Jerònia Lladó^1,2^, Gabriel Olmos^1,2^, Konstantin Strauch^5,6^, Damià Heine-Suñer^2,4^, Cristòfol Vives-Bauzà^1,2¶*^, Antònia Flaquer^5,6,7¶^.

AFFILIATIONS:

1. Department of Biology, University of Balearic Islands (UIB), Institut Universitari d'Investigacions en Ciències de la Salut (IUNICS)

2. Research Unit, Son Espases University Hospital (HUSE), Health Research Institute of Balearic Islands (IdISBa)

3. Department of Psychiatry, HUSE, IdISBa

4. Department of Genetics (GEN-IB), HUSE, IdISBa

5. Chair of Genetic Epidemiology, IBE, Faculty of Medicine, LMU Munich,

6. Institute of Genetic Epidemiology, Helmholtz Zentrum München-German Research Center for Environmental Health, Neuherberg, Germany.

7. Institute for Medical Information Processing, Biometry and Epidemiology - IBE, LMU, Munich, Germany

^¶^ These authors contributed equally to this work

**Supplementary Information**

**Legends Supplementary Tables and Figures**

**S1 Table. Clinical characteristics of the family subjects.** MDD, Major Depressive Disorder; RMDD, Recurrent Major Depressive Disorder; APE F23, Average Partial Effects Acute Transient Psychotic Disorder; SCZ, Schizophrenia; BD-I, Bipolar Disorder I; ADHD, Attention Deficit Hyperactive Disorder; GAF, Global Assessment of Functioning Score; PANSS, Positive and Negative Syndrome Scale (P: Positive, N: Negative; GP: General Psychopathology).

**S2 Table 2. Suggestive regions of Linkage with LOD scores ≤1 for the Narrow (A) and for the Wide (B) phenotypes**. The coordinates within each chromosome (chr) are based on the human genome reference CRGh37/hg19. *P-values* adjusted by Benjamin-Hochberg and Bonferroni (BH).

**S3 Table. CNVs identified using the *cnv*Partition algorithm.** CNVs detected using the *cnv*Partition algorithm, based on the B allele frequency and the log ratio R (LRR: logarithm of the observed/expected probe intensity). DEL, deletion; DUP, duplication.

**S4 Table. Rare coding variants identified in the suggestive regions of Linkage.** Genomic regions associated with the Narrow phenotype (A); the Wide phenotype (B) or both (C) are shown. Allele frequencies are given according to gnomAD and 1000Genomes (1000G). VUS, variant of uncertain significance; NA, not available.

**S5 Table. Rare SNVs and INDELS present in the haplotype blocks of the Linkage Region 9q33.1-33.2 associated with the wide (A) and narrow (B) phenotypes in the Subfamily 3.** Allele frequencies are given according to gnomAD and 1000Genomes (1000G). VUS, variant of uncertain significance; NA, not available.

**S6 Table.** **Polygenic risk scores for schizophrenia (PRS SCZ), bipolar disorder (PRS BD) and combined (PRS SCZ+BD).** MDD, Major Depressive Disorder; APE F23, Average Partial Effects Acute Transient Psychotic Disorder; SCZ, Schizophrenia; BD-I, Bipolar Disorder I; ADHD, Attention Deficit Hyperactive Disorder. PRS were calculated using *PRSice* software.

**S1 Fig. Family pedigree.** In black, subjects diagnosed with psychosis (includes SCZ, BD-I and APE F23); in orange, subjects with history of mental disorders without psychosis (includes MDD and ADHD); in grey, unknown individuals.

**S2 Fig. inPHAP phased haplotype view for the chromosome 9 performed using SNPs with MAF<30% from SNP array.** Haplotype phasing of the entire chromosome 9, performed using SHAPEIT4 version 4.2 and visualized using inPHAP. A zoom of the linkage and the associated region at 9q33.1-33.2 is shown (dashed rectangle). The phenotypes of each subject are color coded: In black, subjects diagnosed with psychosis (includes SCZ, BD-I and APE F23); in orange, subjects with history of mental disorders without psychosis (includes MDD and ADHD); in white, healthy individuals.

**S3 Fig. inPHAP phased haplotype view for the chromosome 9 performed using SNPs with MAF<0.5% from WGS.** Haplotype phasing of the entire chromosome 9, performed using SHAPEIT4 version 4.2 and visualized using inPHAP. The genetic map of the regions of Association (chr9:121492783-122292842) and the H2 haplotype region associated with the wide phenotype at Subfamily 3 (chr9:117843831- 119629686) are zoomed in. The phenotypes of each subject are color coded: In black, subjects diagnosed with psychosis (includes SCZ, BD-I and APE F23); in orange, subjects with history of mental disorders without psychosis (includes MDD and ADHD); in white, healthy individuals.

**S4 Fig. NPL score results for chromosome 3.** **(A)** NPL score results for Chromosome 3. The -log10 (*P* value) of the family-based association test in regions with significant NPL scores are shown as dark green or light green dots for the wide and narrow phenotype, respectively. **(B)** Regional association plot for the 3q26.31-26.33 linked region.

**S1 Table**

**S2 Table**


**S3 Table**

**
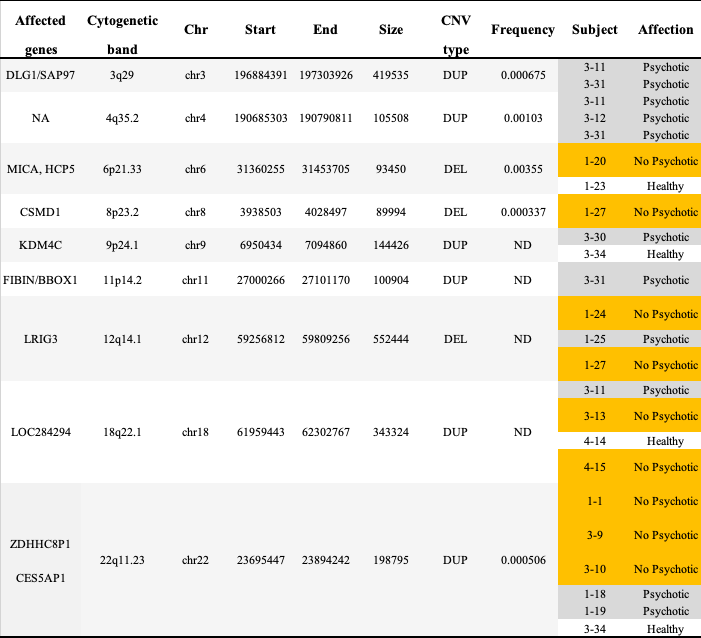
**

**S4 Table**

**S5 Table**

**
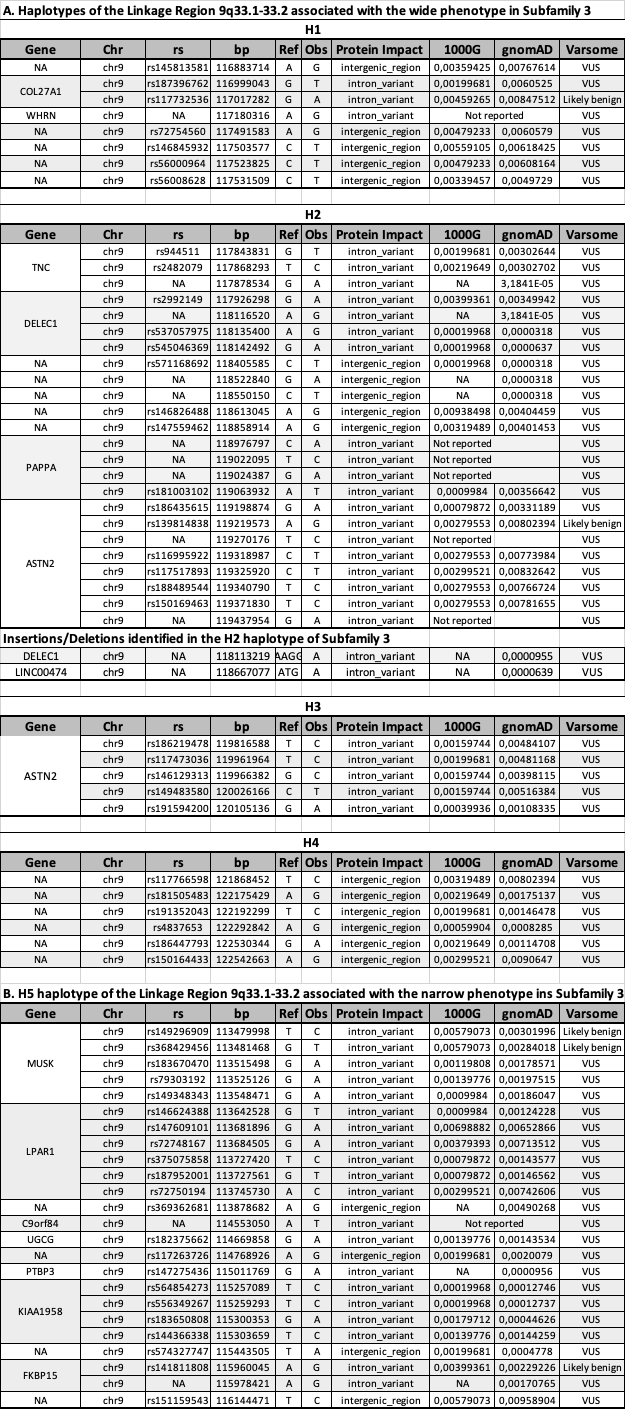
**

**S6 Table**

**
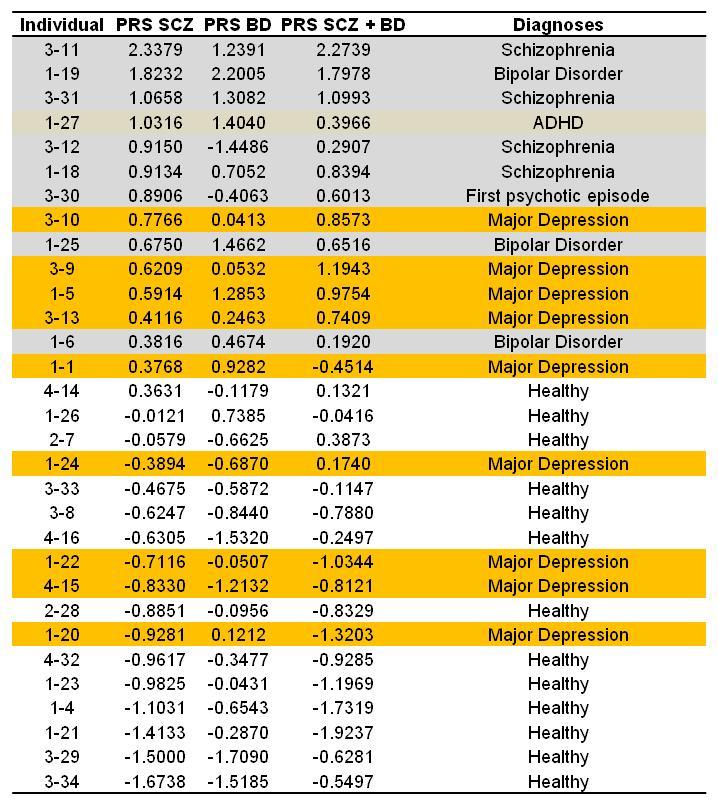
**

**S1 Figure**

**
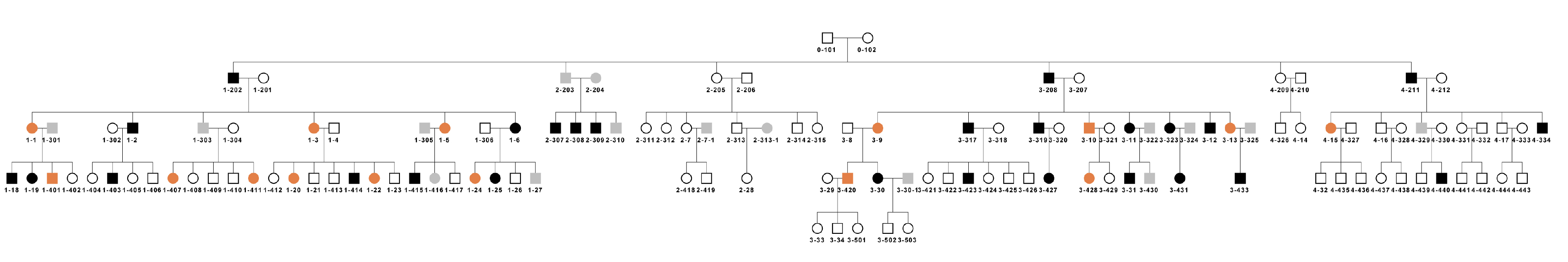
**

**S2 Figure**

**
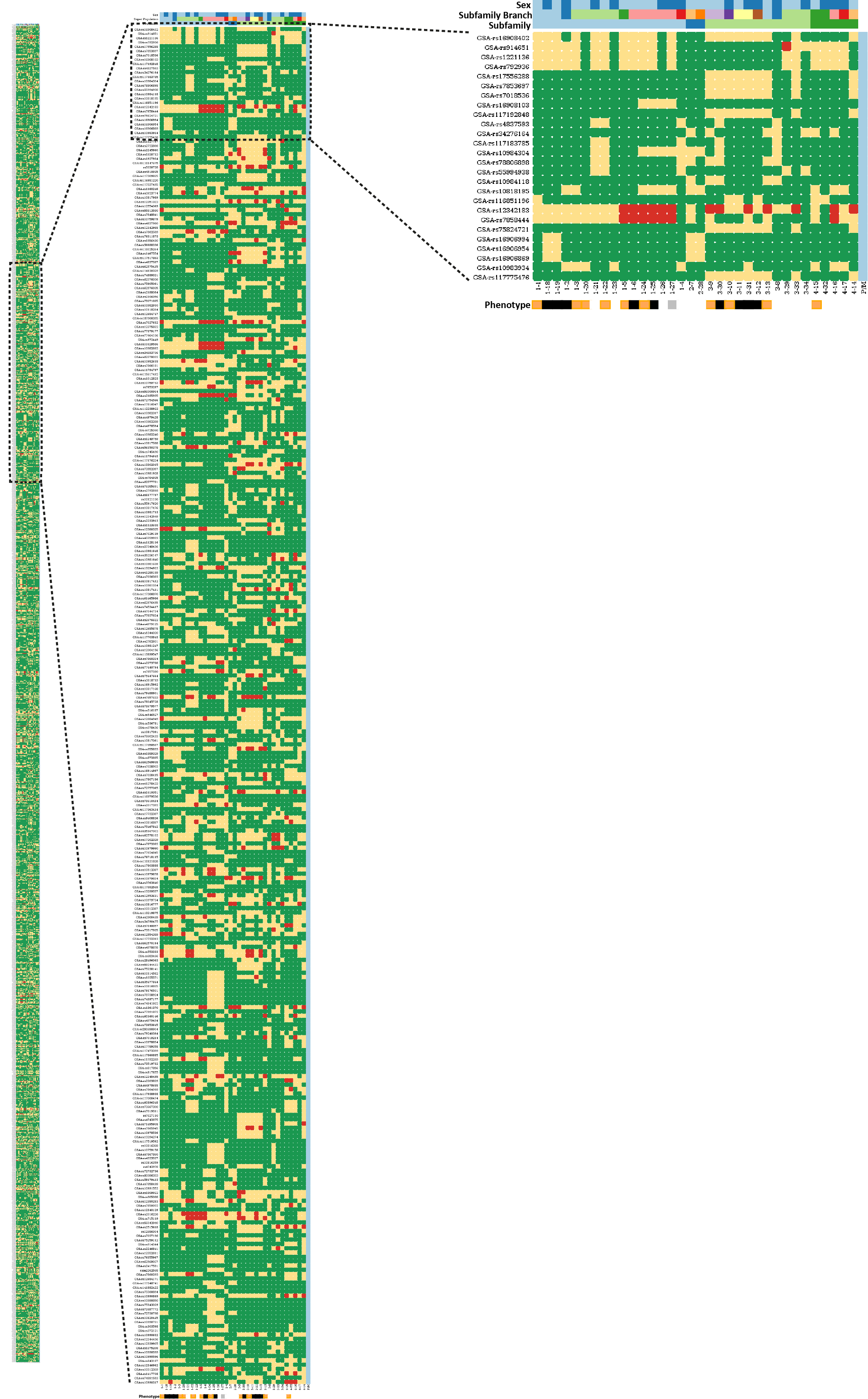
**

**S3 Figure**

**
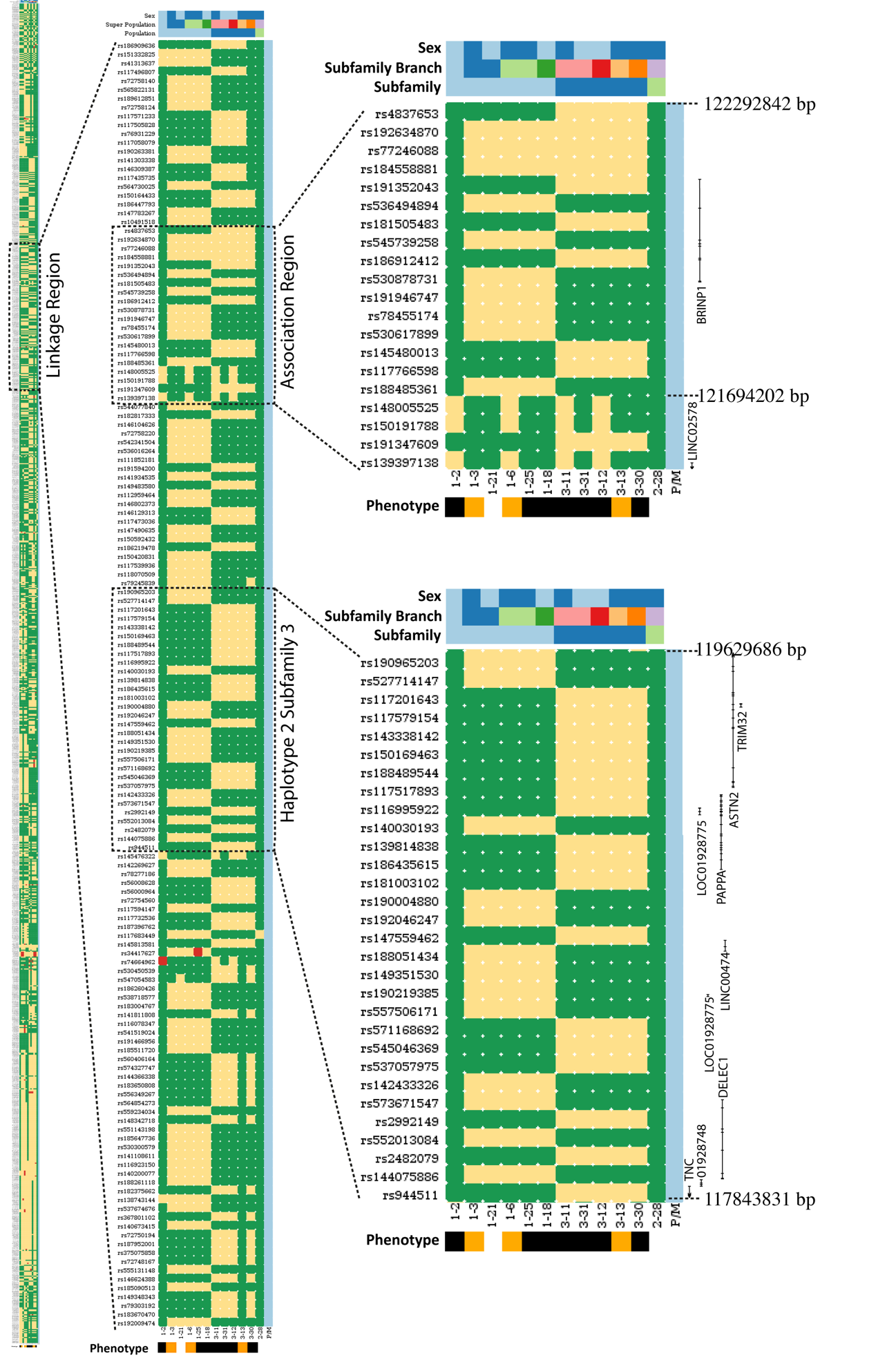
**

**S4 Figure**

**
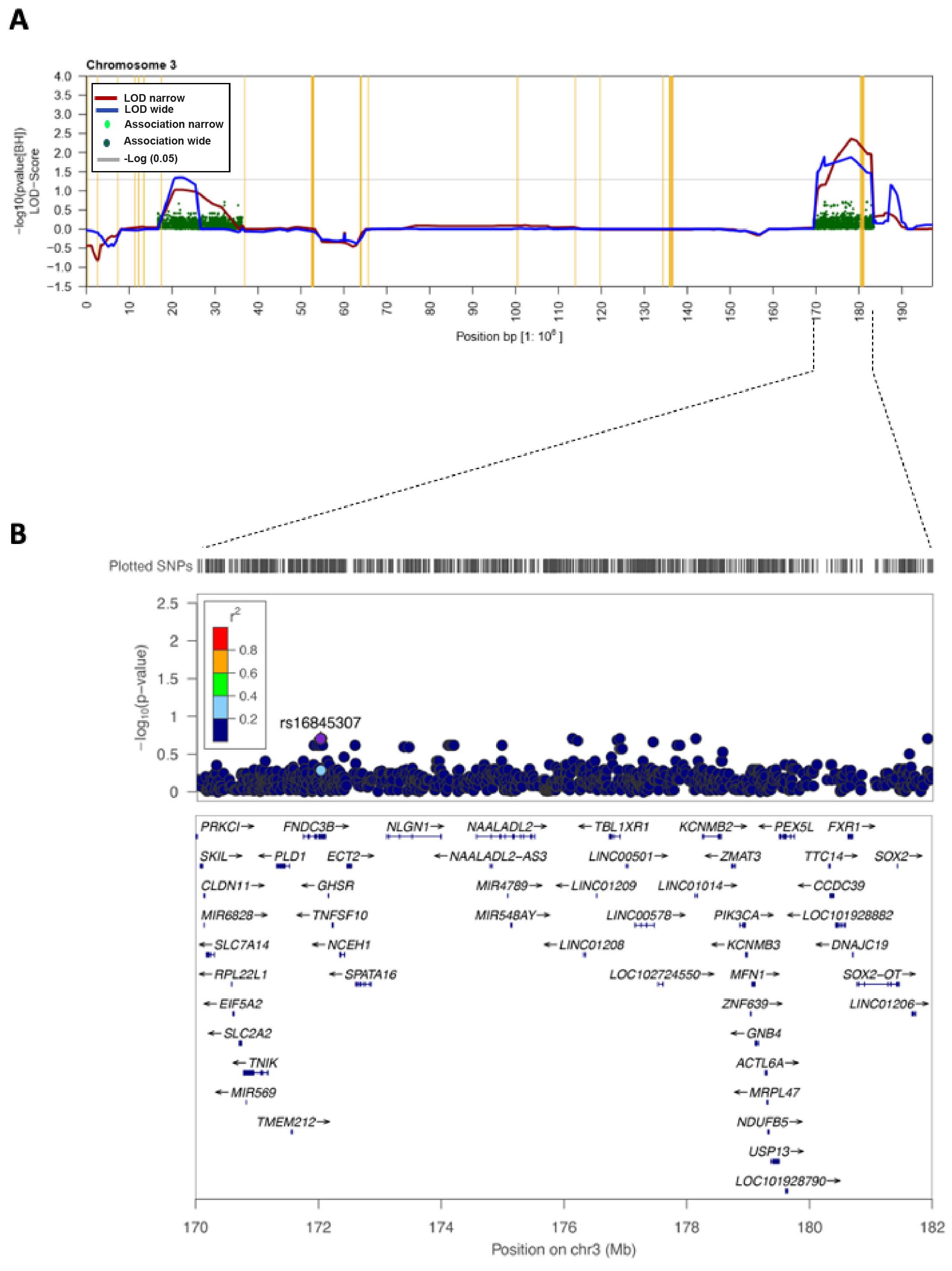
**
